# The Impact of COVID Restrictions Among Rates of Falling in Assisted Living Residents

**DOI:** 10.1093/geroni/igab046.540

**Published:** 2021-12-17

**Authors:** Margaret Danilovich, Melanie Fong, Kamil Hester

**Affiliations:** 1 CJE SeniorLife, Skokie, Illinois, United States; 2 University of Chicago, Chicago, Illinois, United States; 3 Loyola University, Chicago, Illinois, United States

## Abstract

As a result of the COVID-19 pandemic, assisted living group activities and congregate dining stopped and residents were confined to their rooms. While this may have kept residents safer from contracting the virus, it also reduced their physical activity levels. The aim of this study was to investigate if rates of falls in one assisted living community varied as a result of COVID-19 restrictions. We analyzed fall incident reports from n=155 residents from October 2019 to October 2020. Results showed a total of n=802 falls in the year-long period (range of 1-30 falls per resident; mean = 5.17; SD=5.6 in the 12 month period). The majority (65%) of falls occurred in resident rooms. 55% of falls occurred between 6am and 6pm. The primary cause of these falls was loss of balance (30%). Comparing falls that occurred 5 months pre-restriction (Oct 2019-Feb 2020) with 5 months post-restriction (April 2020-August 2021) showed non-significant differences between time periods (p=.59). However, analyzing rates of falls by month showed a range of 46 - 88 falls by month with the lowest number occurring in winter months and the peak number of falls occurring in both Oct 2019 and 2020. Despite the majority of falls occurring in resident rooms, Covid restrictions of room confinement did not appear to impact the prevalence of falls in this sample. However, the seasonal variation warrants further research and those in assisted living should consider seasonal variations and proactively implement policies to prevent falls during these times.

